# Geometric and dosimetric evaluation of CTV contour adaptations by radiation therapists for adaptive prostate radiotherapy on a 0.35 T MR-Linac

**DOI:** 10.1016/j.tipsro.2025.100302

**Published:** 2025-01-13

**Authors:** Boaz Kalkhoven, Marjolein N. Hilberts, Melissa A.L. Verdonk, An-Sofie E. Verrijssen, Peter-Paul G. van der Toorn, Tom C.G. Budiharto, Patricia F.C. Bronius, Diana Geerts, Coen W. Hurkmans, Shyama U. Tetar, Rob H.N. Tijssen

**Affiliations:** a Dept of Radiation Oncology, Catharina Hospital Eindhoven, Michelangelolaan 2, 5623 EJ Eindhoven, the Netherlands; b Dept of Electrical Engineering, Technical University Eindhoven, Groene Loper 19, 5612 AP Eindhoven, the Netherlands; c Dept of Applied Physics and Science Education, Technical University Eindhoven, Groene Loper 19, 5612 AP Eindhoven, the Netherlands; d Dept of Biomedical Engineering, Technical University Eindhoven, Groene Loper 5, 5612 AE Eindhoven, the Netherlands

**Keywords:** MR-Linac, Prostate radiotherapy, Radiation therapist, Contour adaptation

## Abstract

**Background & purpose:**

Magnetic resonance guided adaptive radiotherapy (MRgART) enables hypofractionated prostate radiotherapy by daily contour adaptation. The MRgART workflow, however, is labour intensive and in many institutes still requires the presence of the radiation oncologist (RO). Transferring the online contour adaptation task to the radiation therapist (RTT) will release the clinician from attending each treatment fraction making MRgRT more efficient and cost effective. In this study we investigate the viability of RTT-led prostate MRgART on a low-field MR-linac, by assessing the interobserver variations of RTT- and RO-generated CTV contour adaptations as well as the resulting dosimetric effects.

**Materials & methods:**

Four RTTs and four ROs performed CTV contour adaptations on first fraction data in ten patients. Delineations were compared against a gold standard contour using target volume, Dice similarity coefficient (DSC), and 95th percentile Hausdorff distance. In addition, a dosimetric evaluation was performed on all first fractions by performing plan adaptations based on all RTT contour adaptation and comparing these to the clinically delivered plan. Finally, a full-treatment simulation was performed in four patients to investigate the dosimetric effects of the RTTs’ contour adaptations throughout an entire treatment.

**Results:**

RTTs with no experience in prostate delineation prior to this study spent more time on CTV contour adaptations. The geometric and dosimetric analyses, however, showed no statistically significant differences between both groups.

**Conclusions:**

This study confirmed that RTTs perform similarly to ROs in carrying out online contour adaptations. These results indicate the feasibility of initiating a transition in contour adaptation tasks from ROs to RTTs.

## Highlights

• Inter-observer variations of contour adaptations by radiation therapists were compared to those of radiation oncologists.
• Differences in inter-observer variations and comparison to gold standard contours were statistically insignificant.
• Contour adaptations by radiation therapists had minimal dosimetric effects.

## Introduction

Magnetic resonance guided online adaptive radiation therapy (MRgART) has shown great promise in accurate delivery of ultra-hypofractionated, stereotactic prostate radiotherapy, with many centres now treating low and intermediate-risk prostate carcinoma in 5 fractions (5 x 725 cGy) [1], [2]. MRgART enables reduced treatment margins due to the excellent soft tissue contrast, gated treatment delivery, and the ability to adapt each treatment plan to the daily anatomy, which results in reduced toxicity compared to non-adaptive cone beam guided treatments [3]. MRgART with daily plan adaptation, however, is a resource intensive and time consuming process, which requires the presence of the entire clinical team [4]. Traditionally, the radiation oncologist (RO) is present for each fraction as contouring, specifically tumour or target volume, is traditionally the responsibility of the clinician. In order to create a more time-efficient and cost-effective treatment, several studies have explored physician-free, RTT driven MRgART workflows, in which the contour adaptation task is delegated to the RTT [5], [6], [7], [8], [9], [10], [11].

From these studies most describe the early experience of the clinical implementation [5], [7], [8], [11], and report on quantitative and qualitative comparisons between therapist's and oncologist's contours. A few analysed contouring performances of multiple ROs and RTTs before clinical implementation [6], [9]. All these studies, however, are performed on 1.5 Tesla MRI. At present, one study has investigated the contouring performance of RTTs on low field (0.35 Tesla) MRI [10], but the study by Ricci et al. only focused on organ at risk (OAR) delineation. To the best of our knowledge, the accuracy of target contour adaptation on a 0.35 T MR-Linac has not yet been assessed.

In this study an encompassing geometric and dosimetric analysis is conducted to test the feasibility of an RTT-driven stereotactic prostate MRgART treatment in five fractions. First, the interobserver variation of prostate CTV contour adaptation was assessed for both RTTs and ROs by performing an offline workflow simulation, which simulated the first fraction of 10 previously treated patients. In addition, the dosimetric effect of the RTT contour adaptations was assessed by comparing the RTT-contoured treatment plans to the clinically delivered treatment plans. Finally, in four patients the remaining four fractions were simulated and contoured by all RTTs to evaluate the dosimetric effect on the entire (five fraction) treatment course.

## Methods

### Prostate MRgART protocol and workflow

Patients with low and intermediate risk prostate carcinoma that are treated in a curative MRgART setting receive 5 fractions of 7.25 Gy per fraction on a 0.35 T MRIdian MR-Linac (ViewRay Inc., Oakwood, USA) in our institution. The clinical target volume (CTV) is delineated following the ESTRO ACROP consensus guidelines [12]. For ‘low risk’ patients, the CTV consists of the prostate gland, while for ‘intermediate risk’ patients, the base of the seminal vesicles is also included in the CTV. Contour adaptation of the CTV and surrounding OARs is performed for every fraction. During treatment delivery exception gating is performed based on (single slice) sagittal cine MRI and automatic tracking of the target. Because of the daily adaptation in combination with exception gating a small isotropic planning target volume (PTV) of 3 mm surrounding the CTV is used.

The initial (baseline) treatment plan is optimized using 21 beam step-and-shoot intensity modulated radiotherapy (IMRT) approach. Required PTV coverage is set to V95% > 98 % (other target goals and dose constraints are listed in Table A1).

Each treatment fraction the contours form the baseline plan are propagated onto the daily 3D MRI scan. OARs are propagated deformably, while the CTV is propagated rigidly to better visualize the physicians intend. Manual contour adaptation of the CTV is thus nearly always required. To aid the recontouring process the operator is able to review the baseline MRI and contours on a second screen next to the online workstation.

### Patient data

For this study, data from ten randomly selected patients with localised prostate carcinoma who received stereotactic MRgART between July 2022 and December 2022 at our department were retrospectively collected. These patients were treated with 5 fractions of 7.25 Gy on the MR-Linac. The MR images used for this analysis were 0.35 T TRUFI (true fast imaging with steady-state free precession) images with an isotropic resolution of 1.5 x 1.5 x 1.5 mm^3^. MR data was combined with relevant clinical data (i.e., T-stage, PSA-level, Gleason-score, and tumour localisation), anonymised, and made available for recontouring. Since all patient data were retrospectively collected and fully anonymised, the requirement to obtain informed consent was waived according to the Dutch Medical Research Involving Human Subjects Act.

### Simulated online workflow

CTV contour adaptations were performed by four ROs and four RTTs in a simulated MRgART workflow using the MRIdian ViewRay software. The four ROs participating in this study all had a minimum of 12 months of experience with the online MRgART workflow and performing contour adaptations for prostate cancer patients. The four RTTs participating in this study had a minimum of 12 months of experience with the online prostate cancer workflow and pre-treatment offline OAR contouring, but not the CTV. At the start of this study, all RTTs underwent a comprehensive training session on CTV contouring, including how to weigh specific tumour characteristics. The training was conducted by one of the ROs with over five years of experience in contouring on 0.35 T images. This training session included both a theoretical lecture and a practical component, during which the RTTs contoured four CTVs and received feedback. The patients discussed in the training session did not overlap with those used in the study.

MR images and original contours of the 10 baseline treatment plan were imported and registered to their first fraction images simulating the online workflow, after which the ROs and RTT performed their contour adaptation. Adaptation time was recorded to simulate the online workflow time-pressure. Contour adaptations were done individually and blinded so that the ROs and RTTs could not look at or compare contours from each other and within their groups.

### Geometric analysis

The interobserver variation in CTV contouring was assessed geometrically to examine the spatial differences in contour adaptations between ROs and RTTs. Furthermore, this analysis aimed to explore the differences between the ROs and RTTs within their respective groups. For this analysis the adapted CTV contours were extracted and processed in Matlab (The MathWorks Inc., R2021b) [13] and the clinically used contours were used as the gold standard contour for all analyses. Contour volumes were calculated and percentage volume differences between the adapted contours and gold standard contours were assessed. Furthermore, the Dice similarity coefficient (DSC) and (95th percentile) Hausdorff distances were calculated in order to quantify differences between the adapted and gold standard contours. Conformity was calculated for both ROs and RTTs versus the gold standard delineation.

### Dosimetric analysis

To assess the dosimetric consequences of variations in contour adaptation, a dosimetric analysis was conducted for the first fraction adaptations made by the ROs and RTTs in all patients. For this analysis a collection of new treatment plans was created based on each adapted CTV by deriving a new PTV and removing potential overlap with the OAR contours as per normal online workflow. The new treatment plans were created using the same (template) objectives and optimization parameters as used in our normal online workflow.

The newly formed, single fraction, treatment plans were then evaluated on the gold standard contours (i.e., the original clinically used CTV and PTV) in order to see if the clinical dose goals would still be met given the geometric differences of the RO and RTT contours.

Clinical goals and dose constraints were assessed on the PTV, CTV and OAR structures. In addition, a CTV + 1.5 mm structure was defined, which is complementary to the CTV evaluation, but includes a small (residual) margin to account for inaccuracies during treatment delivery.

Finally, in order to explore the dosimetric effects of the RTTs' adaptations on a full treatment course, fractions 2–5 were also simulated and adapted by all RTTs in four patients. This full treatment dosimetric analysis entailed the same steps as explained previously, repeated for every simulated fraction. The patients were selected following the initial dosimetric results and included two patients categorized as “typical” and two designated as “challenging”. The overall dosimetric effects of the full treatment course were determined by calculating the mean of the DVHs across all simulated subsequent fractions per patient.

**Fig. 1:**
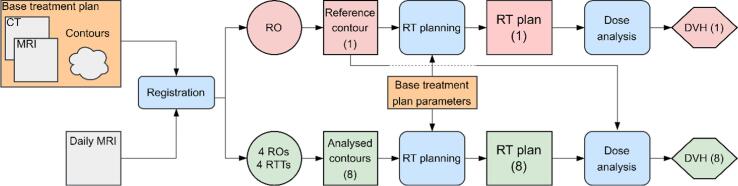
Example of the simulated online workflow analysis for generating a treatment plan on the RO- and RTT-generated contours (green flowchart symbols) and evaluating on the reference contours (red flowchart symbols). (For interpretation of the references to colour in this figure legend, the reader is referred to the web version of this article.)

A full overview of the dosimetric analysis workflow is shown in Fig. 1. For all dosimetric analyses, dose values and DVHs were recalculated in and extracted from the SlicerRT toolkit [14] within 3DSlicer (slicer.org) [15] and analysed in Matlab (The MathWorks Inc., R2021b) [13].

### Statistical analysis

Wilcoxon rank sum tests were used to test for significance in differences between the RO and RTT group. This test was performed for every metric at a statistical significance level of 5 % (p < 0.05).

## Results

### Interobserver variability

**Fig. 2:**
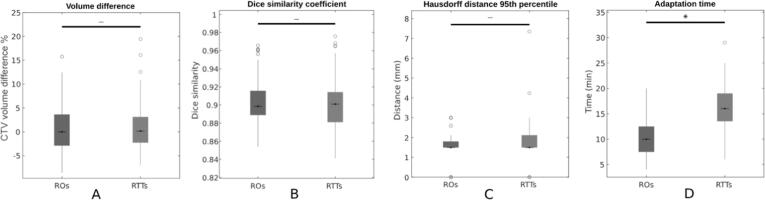
Geometric analysis results for RO- and RTT-generated CTV contours compared to the gold standard contours. Percentage volume differences (A), Dice similarity coefficient (B), 95th percentile Hausdorff distance (C) and contour adaptation time (D). Statistically significant differences marked with * (p < 0.05).

Results for the percentage volume difference, DSC, 95th percentile Hausdorff distances, and adaptation time are shown in Fig. 2. The median and interquartile range (IQR) contour adaptation time was 10 (7.25–12.75) minutes for the RO and 16 (13.23–19) minutes for RTT group. This difference between the two groups was statistically significant (p < 1 x 10^-7^). Visual inspection of the generated contours showed that the interobserver variability was largest around the vesicles and at the apex of the prostate. The median (IQR) percent volume difference for the ROs was 0.00 % (−2.90 % − 3.63 %) and for the RTTs was 0.15 % (−2.27 % − 3.12 %). The median (IQR) DSC was 0.90 (0.89–0.92) for the ROs and 0.90 (0.88–0.91) for the RTTs. The median (IQR) 95th percentile Hausdorff distance being 1.5 mm (1.50 mm − 1.81 mm) for the ROs and 1.5 mm (1.50 mm − 2.12 mm) for the RTTs, with the maximum Hausdorff distances observed around 16 mm for both groups. The resulting differences in these metrics between the two groups did not reach statistical significance.

### Single fraction dosimetric effects

An overview of the single fraction results over all patients is shown in Table 1. Overall, differences between the RO and RTT delineated treatment plans were very small and non-significant with p-values of 0.90 and 0.86 for the CTV and PTV analysis, respectively. The median V95% of the PTV was 97.3 % for the plans based on the RO contours and 97.73 % for the treatment plans based on the RTT contours. The median V95% CTV coverage is shown to be over 99.98 % for both groups. Finally, the *intermediate* CTV + 1.5 mm structure showed a median V95% of a least 99.6 %, for both groups. Two out of ten patients proved to be “challenging” as for some optimized plans within both the RO and RTT groups, the V95% PTV dose coverage was below 90 %.

**Table 1:** First fraction dosimetric results over all patients; evaluated gold standard structure, contours on which the treatment plan was optimized and median volume that received 95% of the prescribed dose.

**Structure**	**Evaluated plan**	**Median V95% [IQR]**
PTV	Clinical plan Fr1	99.00 % [98.64–99.11]
PTV	RO contoured	97.32 % [96.00–98.59]
PTV	RTT contoured	97.73 % [95.63–98.56]
CTV	Clinical plan Fr1	100 % [99.99–100]
CTV	RO contoured	99.98 % [99.90–100]
CTV	RTT contoured	99.99 % [99.87–100]
CTV + 1.5 mm	RO contoured	99.66 % [99.23–99.92]
CTV + 1.5 mm	RTT contoured	99.74 % [98.86–99.93]

**Fig. 3:**
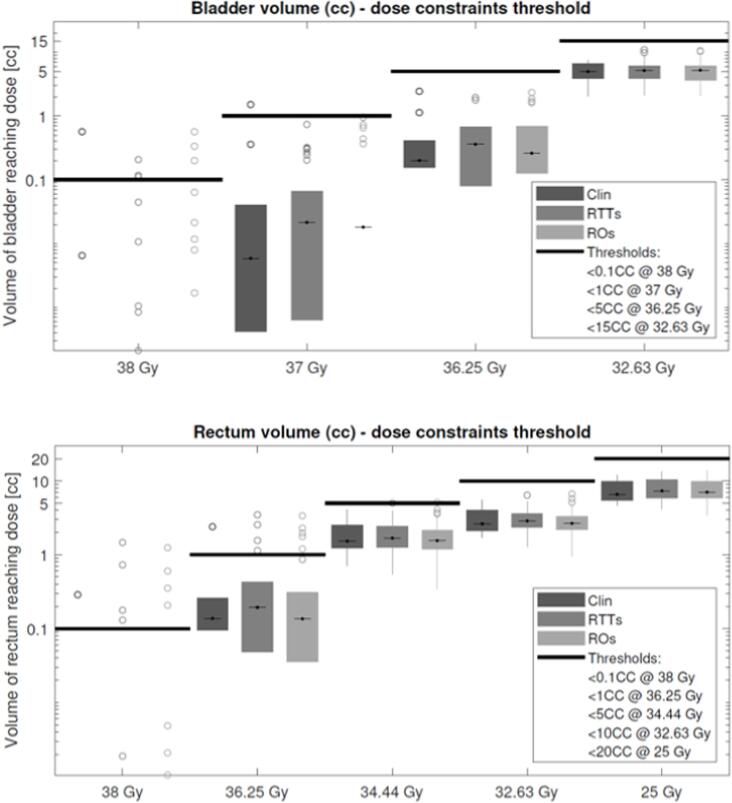
The absolute volumes (cc) of the bladder and rectum receiving the various dose constraint values for the clinically delivered, the RTTs', and the ROs' plans. The maximum volumes allowed are denoted by the black lines. Boxes denote the 25th and 75th percentile, while the whiskers include all data points not considering outliers. Outlier data points are represented by ‘o’. Boxes at 38 Gy are absent because the vast majority of plans had 0 cc volume dose coverage at 38 Gy. Clinical plans' boxes contain 10 (1 clinical plan for 10 patients) datapoints. RTTs' and ROs' boxes contain 40 (4 plans for 10 patients) datapoints.

The results of the OAR dosimetric analysis over all patients are shown in Fig. 3. Dose constraints were met by the vast majority of the ROs' and RTTs' plans. In a few plans, however, the bladder and rectum constraint for the higher dose levels was exceeded. The treatment plans that violated the dose constraints for both the ROs and RTTs were confined to one specific patient. In this single case, the clinically used plan also surpassed high dose level (38 Gy and 36.25 Gy) rectum constraints. For the other nine patients, all OAR dose constraints were successfully achieved by all RO and RTT contoured treatment plans.

### Full treatment course dosimetric analysis

**Fig. 4:**
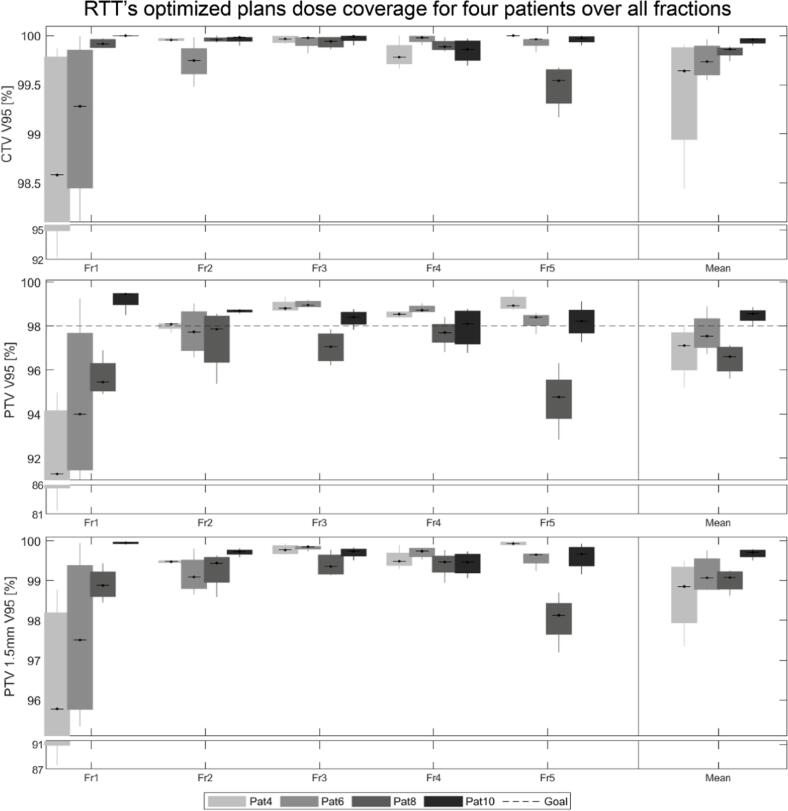
Full treatment course analysis in four selected patients. V95% coverage evaluated on the gold standard CTV (top), PTV (middle) and PTV 1.5 mm (bottom) structures over all fractions and for all four patients. The mean V95% results over all fractions (right) are shown on the far right of the plot. The dotted line in the centre figure indicates our institute’s clinical goal on the PTV (V95% > 98 %). Note: y-axes are broken up for better visualisation.

The results of the full treatment dosimetric analysis in two “challenging” (Pat4, Pat6) and two “typical” (Pat8, Pat10) patients are shown in Fig. 4. It is seen that the dose coverage of the first fraction of Pat4 and Pat6 (classified as challenging) is worse than the coverage in Pat8 and Pat10. For the subsequent fractions (fraction 2–5), however, the dose coverage is higher compared to fraction 1 and similar to the coverage obtained in Pat8 and Pat10. Pat8 shows a reduced coverage in fraction 5 compared to the other four fractions in this patient. Overall, for the majority of the fractions and across all patients the PTV V95% was over 95 % and the CTV V95% over 99.5 %. The CTV + 1.5 mm margin coverage was close to 99 %, which is also reflected by the mean dose coverage plotted on the far right of Fig. 4. The median V95% dose coverage results over all four patients over all five fractions are shown in Table 2.

**Table 2:** Full treatment course dosimetric results over four patients including two typical and 2 challenging cases; evaluated gold standard structure, contours on which the treatment plan was optimized and median volume that received 95% of the prescribed dose. V95% values were calculated by taking the mean over all five fractions.

**Structure**	**Evaluated plan**	**Median V95% [IQR]**
PTV	Clinical plans Fr1-5	98.92 % [98.79–98.99]
PTV	RTT contoured	98.37 % [96.87–98.75]
CTV	Clinical plans Fr1-5	100 % [99.98–100]
CTV	RTT contoured	99.96 % [99.83–99.99]
CTV + 1.5 mm	RTT contoured	99.56 % [98.15–99.81]

## Discussion

The main goal of this study was to investigate whether the role of online contour adaptation of the prostate CTV, currently performed by the RO, can be transferred to the RTT using the initial (baseline) contours as a guidance. Our geometric analysis showed that the interobserver variation of the RTT group and RO group was comparable and both groups demonstrated similar CTV volume differences, DSC and Hausdorff distances compared to the gold standard contour. No statistical differences were found when comparing both groups, except for delineation time. The DSC values of around 0.9 found in this study are comparable to earlier work performed on a 1.5 T MR-linac [5], [6], [7], [9] indicating that for both field strengths the level of agreement is similar for both the RTT and RO group. The median contouring times of the RTT group was 16 min, which is longer than previously reported by others (around 7 min in [7] and 12 min in [5], [11]). Note, however, that the RTT group had no experience in CTV delineation at the start of this study. It is assumed that additional training and will reduce delineation times to the level of ROs as also shown by others [7], [16]. This will be evaluated after clinical implementation in our department.

The single fraction dosimetric analysis (Table 1) showed that the dose coverage for the RO and RTT contoured treatment plans were in good agreement with the original clinical treatment plans. When evaluating the V95% of the clinically used (i.e., gold standard) PTV contour on the treatment plans that were optimized based on either the new RO or RTT contour adaptations, we found that a median V95% > 97 %, indicating that the interobserver variations had minimal dosimetric effects. Because our PTV V95% > 98 % is reasonably high compared to the PTV V95% > V95% reported by others [2], all treatment plans created with RTT contours achieved PTV V95% > 95 %, and were therefore considered clinically acceptable. This finding is in line with previous studies [5], [11]. In addition, the analysis of the PTV with a smaller margin (CTV + 1.5 mm) showed that even the stricter PTV goal (PTV V95% > 98 %) can be met while keeping a margin for residual uncertainties during dose delivery. Note, however, that the 1.5 mm margin stems from the fact that the treatment planning software only allows PTV expansions that are a multiple of the imaging resolution (i.e., steps of 1.5 mm) and is not based on a proper margin assessment. Further research would be needed to assess whether the 1.5 mm CTV expansion fully covers all geometric uncertainties during dose delivery.

To explore the effects of the RTTs' contour adaptations on a full, five-fraction, treatment course, the dosimetric analysis was repeated over all fractions in four patients. Among these four patients, two *challenging* patients and two *typical* patients were selected based on the first fraction results. The main reason for selecting the two challenging patients was to simulate a worst case scenario. The fact that fraction 1 presented larger deviation was attributed to the sub-optimal image quality of the images for that fraction. Something which was confirmed by the RO group upon retrospective inspection. The full treatment course analysis, however, showed that the average coverage was clinically acceptable as depicted by the CTV + 1.5 mm results. This can be attributed to the fact that with online MRgART systematic delineation errors become more random in nature [17]. In this simulation study, the RTTs and ROs were not allowed to ask assistance in adapting the contours. In practice, this would be allowed potentially minimizing such outlier occurrences in more challenging patients.

A general limitation of this type of contouring study is the lack of a ground truth delineation. In this study a gold standard contour was defined as the expert contour that was used clinically. Using the clinically used CTV contour as a gold standard allowed a direct comparison of the simulated treatment plans with the clinically delivered treatment. In the past, others have used the average contour of the clinicians [6], but this introduces a bias towards the clinicians’ contours, which we aimed to avoid as much as possible. The patients in this study were randomly selected, which raises a minor concern that a radiation oncologist (RO) may have contoured a case previously. A minimum 7-week interval between clinical treatment and the start of the study was therefore introduced to mitigate this potential bias.

The analyses conducted in this study revealed that, in terms of both geometric and dosimetric aspects, there are no statistically significant differences between the prostate cancer contour adaptations carried out by ROs and RTTs. The number of patients used in this study is, however, limited. A secondary evaluation after clinical implementation, similar to [5] is therefore warranted to confirm the results presented here. A careful evaluation would also be required when the RTT-led MRgART workflow is expanded to other tumour sites.

## CRediT authorship contribution statement

**Boaz Kalkhoven:** Software, Formal analysis, Validation, Writing – original draft, Visualization. **Marjolein N. Hilberts:** Data curation, Formal analysis, Writing – original draft. **Melissa A.L. Verdonk:** Data curation, Formal analysis, Writing – original draft. **An-Sofie E. Verrijssen:** Data curation. **Peter-Paul G. van der Toorn:** Data curation. **Tom C.G. Budiharto:** Data curation. **Patricia F.C. Bronius:** Data curation. **Diana Geerts:** Data curation. **Coen W. Hurkmans:** Methodology, Writing – review & editing. **Shyama U. Tetar:** Conceptualization, Methodology, Supervision, Writing – review & editing. **Rob H.N. Tijssen:** Conceptualization, Methodology, Writing – review & editing, Supervision, Project administration.

## Declaration of competing interest

The authors declare that they have no known competing financial interests or personal relationships that could have appeared to influence the work reported in this paper.

## Figures and Tables

**Table A1:** Dose objectives and constraints.

**Organ**	**Goal / constraint**
**PTV**	V95% > 98 %
**PTV**	V107% < 2 %
**Rectum**	V38Gy < 0.1 cc
**Rectum**	V36.25 Gy < 1 cc
**Rectum**	V34.44 Gy < 5 cc
**Rectum**	V32.63 Gy < 10 cc
**Rectum**	V25Gy < 20 cc
**Bladder**	V38Gy < 0.1 cc
**Bladder**	V37Gy < 1 cc
**Bladder**	V36.25 Gy < 5 cc
**Bladder**	V32.63 Gy < 15 cc
**Small bowel**	V35Gy < 0.1 cc
**Small bowel**	V33Gy < 1 cc
**Small bowel**	V26Gy < 5 cc
**Femoral heads**	V30Gy < 10 cc
